# Validating stroke-induced bilateral ankle coordination deficits using bilateral ankle measure relationship with motor functions in lower limbs

**DOI:** 10.1186/s12984-023-01157-0

**Published:** 2023-03-17

**Authors:** Jia-Lan Chang, Hung-Ju Chen, Po-Yin Chen, Li-Wei Chou, Chien-Hung Lai, Yueh-Hsun Lu, Shang-Lin Chiang, Chia-Huei Lin, Xin-Miao Wang, Chueh-Ho Lin

**Affiliations:** 1 Department of Physical Medicine and Rehabilitation, Shuang Ho Hospital, Taipei Medical University, No. 291, Jhongjheng Rd., Jhonghe, New Taipei, 23561 Taiwan; 2 Master Program in Long-Term Care, College of Nursing, Taipei Medical University, No. 250 Wu-Xing Street, Taipei, 110 Taiwan (R.O.C.); 3 National Center for Geriatrics and Welfare Research, National Health Research Institutes, 35 Keyan Rd., Zhunan Town, Miaoli County, 350 Taiwan (R.O.C.); 4 Department of Physical Therapy and Assistive Technology, National Yang Ming Chiao Tung University, No. 155, Sec. 2, Linong Street, Taipei, 112 Taiwan (R.O.C.); 5 Department of Physical Medicine and Rehabilitation, School of Medicine, College of Medicine, Taipei Medical University, No. 250 Wu-Xing Street, Taipei, 110 Taiwan (R.O.C.); 6 Department of Radiology, Shuang-Ho Hospital, Taipei Medical University, No. 291, Zhongzheng Rd., New Taipei City, 235 Taiwan (R.O.C.); 7 Department of Radiology, School of Medicine, College of Medicine, Taipei Medical University, No. 250 Wu-Xing Street, Taipei, 110 Taiwan (R.O.C.); 8 Department of Physical Medicine and Rehabilitation, Tri-Service General Hospital, School of Medicine, National Defense Medical Center, No. 325, Sec. 2, Chenggong Rd., Taipei, 114 Taiwan (R.O.C.); 9 Department of Nursing, Tri-Service General Hospital; School of Nursing, National Defense Medical Center, No. 325, Sec. 2, Chenggong Rd., Taipei, 114 Taiwan (R.O.C.); 10 Faculty of Humanities, Zhejiang Dong Fang Polytechnic College, 47Th Floor, China Resources Building B 1366 Qianjiang Road, Hangzhou, China; 11 Research Center in Nursing Clinical Practice, Wan Fang Hospital, Taipei Medical University, No. 111, Sec. 3, Xinglong Rd., Taipei, 116 Taiwan (R.O.C.); 12 International Ph.D. Program in Gerontology and Long-Term Care, Taipei Medical University, No. 250 Wu-Xing Street, Taipei, 110 Taiwan (R.O.C.); 13 Department of Physical Medicine and Rehabilitation, Taipei Medical University Hospital, No. 252 Wu-Xing Street, Taipei, 110 Taiwan (R.O.C.)

**Keywords:** Coordination control, Movement disorder, Stroke, Ankles

## Abstract

**Background:**

Coordinated control between the bilateral ankle joints plays an important role in performing daily life functions, such as walking and running. However, few studies have explored the impact of stroke on movement disorders that decrease the coordination control of the bilateral extremities and may decrease daily activities that require coordination control of the bilateral ankles. This study aimed to investigate the coordination control of the bilateral ankles using a novel bilateral ankle measurement system and evaluate the relationship of bilateral movement coordination control deficits with motor and functional performances of the lower extremities in patients with stroke.

**Methods:**

Twenty-one healthy adults (36.5 ± 13.2 y/o) and 19 patients with chronic stroke (58.7 ± 10.5 y/o) were enrolled. A novel measurement device with embedded rotary potentiometers was used to evaluate bilateral ankle coordination control. Participants were asked to move their dominant (non-paretic) foot from dorsiflexion to plantarflexion position and non-dominant (paretic) foot from dorsiflexion to plantarflexion position (condition 1) simultaneously, and vice versa (condition 2). Alternating time and angle for coordination control with movements of both ankles were calculated for each condition. Motor and functional performance measurements of the lower extremities included the lower-extremity portion of the Fugl-Meyer assessment (FMA-LE), Berg Balance Test (BBS), Timed Up and Go Test (TUG), and Barthel Index (BI).

**Results:**

Compared with the healthy group, alternating time was shorter in the stroke group by 8.3% (p = 0.015), and the alternating angles of conditions 1 and 2 were significantly higher than those of the healthy group by 1.4° (p = 0.001) and 2.5° (p = 0.013), respectively. The alternating angle in condition 2 showed moderate correlations with TUG (r = 0.512; p = 0.025), 10-m walk (r = 0.747; p < 0.001), gait speed (r =  − 0.497 to − 0.491; p < 0.05), length (r =  − 0.518 to − 0.551; p < 0.05), and BI (r =  − 0.457; p = 0.049).

**Conclusion:**

Stroke decreases alternating time, increases alternating angle, and shows bilateral ankle coordination control deficits temporally and spatially. A higher alternating angle is moderately to highly associated with motor function and lower limb function in patients with stroke.

## Background

Coordination control and performance in bilateral ankles play important roles in daily activities, such as walking and balance, requiring reciprocal dorsiflexion and plantarflexion movements (ankle rocks) of the bilateral ankles [1–3]. However, stroke leads to muscle weakness, poorer perception, and spasticity of the paretic lower limbs [4–8], which can result in motor and functional deficits and abnormal compensation during walking after stroke [9, 10]. In addition, recent studies reported that the function of unaffected limbs could also be impacted in patients with stroke [11, 12], which may affect the coordination-related functions of both paretic and non-paretic limbs. Meanwhile, many studies have also shown that functional deficits of paretic ankle joints in the lower limbs worsen with increasing time after stroke [13, 14]. This can cause more deterioration impacts on motor and coordination performances between bilateral lower limbs and may increase the dependence on daily care for 1/4–3/4 stroke patients [15]. A recent study also reported that coordination control of ankles could be related to walking performance of patients with stroke [16]. Therefore, appropriate evaluation tools for the direct measurement of coordination control deficits of the movements in both ankles are important in the clinic. These tools could allow assessment of the functional status of bilateral ankle cooperation control and enable the development of appropriate interventions to improve coordination deficits in both ankles and relevant functions in the lower limbs of stroke patients.

The performance of bilateral lower limb coordination is strongly related to the locomotion of patients with neurological deficits, but the current assessments do not seem to completely meet the demand for evaluating bilateral coordination control. For example, the heel to shin coordination test of the Fugl-Meyer Assessment (FMA) was developed and has been commonly applied to quantitatively evaluate the coordination control of bilateral lower limbs of patients with dysmetria and tremors, as well as stroke patients with inaccurate coordination of the lower limbs [17, 18]. The Lower Extremity Motor Coordination Test (LEMOCOT) is also an effective measurement tool with excellent validity and reliability [19], which can be applied to evaluate coordination deficits in the lower limbs [20], detect changes in motor coordination [21] and predict the prognosis of functional recovery [22] in patients with stroke. These tests measure coordination control of the ankle and lower limbs by calculating the repetitions required to complete tasks using the ankle and lower limbs simultaneously, which may indicate the dexterity of paretic lower limbs rather than directly reflect the performance of reciprocal coordination of ankle or lower limbs. Therefore, these assessment tools could not directly determine changes in the movements in coordination control between two ankles while simultaneously executing tasks with both ankles.

Combined biosensors and computer programs have been developed in recent years which can be used to quantify coordination control among the limbs. For example, recent studies employed an evaluation system with two dynamometers to identify that the coordination controls in grip strength between the hands were associated with motor and functional performances in the upper limbs of stroke patients [23, 24]. In contrast, spatial and temporal changes in the movement performance of both ankles during coordination control tasks have rarely been discussed or demonstrated in patients with stroke due to the lack of appropriate assessment tools for both ankles. Furthermore, few studies have investigated the relationship between the time and movement performance of both ankles during coordination control, and the motor and functional performance of the paretic lower limb. Therefore, the aim of this study was to investigate stroke-related changes in coordination control of movements using a bilateral ankle measurement system and to evaluate the relationship between coordination control of the ankles, and the relationship with motor and functional performances of the paretic lower extremity in patients with stroke.

## Methods

### Aim, design and setting

This study aimed to investigate the coordination control of the bilateral ankles using a novel bilateral ankle measurement system; and evaluate the relationship of bilateral movement coordination control deficits with motor and functional performances of the lower extremities in patients with stroke. This prospective cross-sectional observational study was conducted within the hospital setting.

### Participants

Twenty-one healthy adults (36.5 ± 13.2 y/o) and 19 patients with chronic stroke (58.7 ± 10.5 y/o) were invited to participate in this study at hospital. Stroke patients were recruited by clinical specialists in the Department of Rehabilitation Medicine during outpatient visits. Healthy adults were recruited from family members who accompanied the patient to the clinic or from residents of the surrounding community. The inclusion criteria for healthy adults were the absence of disease that would affect the performance of any lower limb movements or foot or ankle dysfunction. For the chronic stroke group, the inclusion criteria were as follows: (1) the stroke event had occurred at least 6 months previously and cardiovascular condition was stable; (2) a unilateral ischemic or hemorrhagic stroke had occurred, as confirmed by collecting each patient’s medical history; (3) the patient was classified as Brunnstrom stage 4 or higher; (4) the patient had a modified Ashworth score of ≤ 3 for the ankle joint and was able to dorsiflex and plantarflex the paretic and non-paretic feet [25]; (5) no other orthopedic or neurologic disorders existed in the lower limbs; (6) the patient had a Mini-Mental State Examination score of ≥ 24 [26]; (7) the patient could sit on a chair and perform coordination control tasks. The exclusion criteria were feeling pain or discomfort during the tasks. Each participant signed an informed consent form before the study. This study was approved by the Joint Institutional Review Board of Taipei Medical University (no. N201904034). The demographic characteristics and clinical motor and functional measurements for patients with stroke are shown in Table 1.

**Table 1:** Demographic data and characteristics of the patients with stroke

Gender	Age (years)	BH (cm)	BW (kg)	On-set time (day)	Lesion area	Type	Lesion side	MMSE	Brunnstrom stage	MAS	FMA-LE	BI	BSS	10 m-walk (s)	TUG (s)
F	55	156	104	419	Putamen	Hemorrhage	Right	30	IV	1+	27	65	29	44.2	29.4
F	48	163	61	344	Basal ganglion	Hemorrhage	Right	30	IV	2	27	70	32	57.4	27.1
M	75	160	47	1482	Thalamic	Hemorrhage	Right	29	IV	1+	25	70	24	95.6	70.8
M	71	154	71	1684	Frontal	Hemorrhage	Left	29	V	1+	28	80	35	20.3	37.6
M	63	161	66	1670	Globus pallidus	Infarction	Left	30	IV	2	25	75	32	78	35.4
M	61	170	60	2100	Basal ganglion	Infarction	Right	30	IV	1	29	95	41	31.9	14.7
F	55	160	48	1870	Thalamic	Hemorrhage	Left	30	IV	1	25	95	34	35.4	13.8
F	45	165	65	693	Basal ganglion	Hemorrhage	Right	30	IV	1+	28	85	36	19.3	15.1
M	57	172	75	3342	ACA	Infarction	Left	30	IV	1+	26	85	35	30.4	23.3
M	63	175	76	1969	MCA	Infarction	Left	29	IV	1+	27	85	40	57.3	32.6
M	47	174	78	253	Putamen	Hemorrhage	Right	29	IV	1+	26	85	30	24.1	19.2
M	77	172	68	967	Thalamic	Infarction	Left	30	V	1	30	90	38	31.5	18.6
F	49	166	63	1343	Putamen	Hemorrhage	Left	30	IV	1+	27	100	39	40.3	27.6
M	46	168	65	882	Putamen	Hemorrhage	Left	30	IV	1+	27	80	38	42.7	20.2
M	72	170	75	1821	Putamen	Hemorrhage	Right	30	IV	2	28	95	44	44	31.2
M	63	166	73	222	Basal ganglion	Infarction	Right	30	V	1	28	95	38	17.9	16
M	47	172	70	267	MCA	Infarction	Left	30	IV	1	32	100	30	14.7	12.4
F	54	158	45	417	Putamen	Hemorrhage	Left	30	IV	1+	27	95	35	18.4	16
M	65	176	86	1484	Thalamic		Right	30	IV	1+	28	100	43	17.5	15.1

*F* female; *M* male; *BH* body height; *BW* body weight; *MMSE* Mini-Mental State Examination; *FMA-LE* Lower limb subscale of the Fugl-Meyer Assessment, *BI* Barth Index, *BBS* Berg Balance Test, *TUG* Time Up and Go

### Measurement device and data processing

**Fig. 1:**
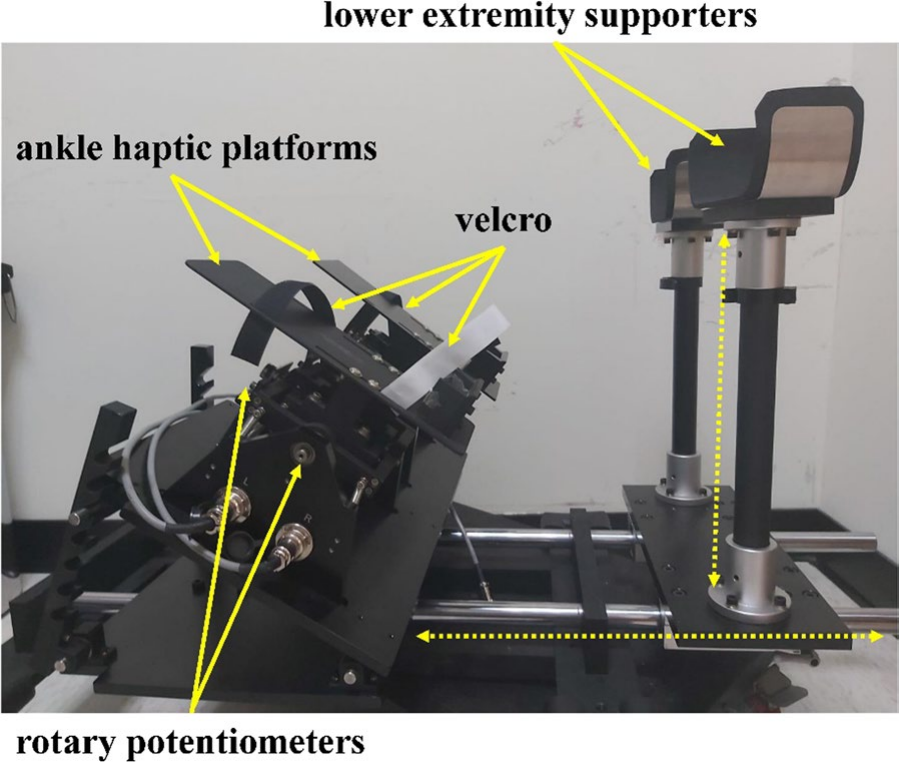
Components of the novel bilateral ankles measurement system

The ankle joint measurement device was developed to measure dual-axis motions in degrees of the ankle joint, and has been shown to have excellent validity [27]. In this study, we used the same material and engineering technologies to develop a novel bilateral ankle measurement system for motions in degrees of the bilateral ankle joints. This system comprised two tilting adjustable ankle haptic platforms with four rotary potentiometers (100 K ± 0.05% W, Rmax, Taipei, Taiwan) (Fig. 1). In the range of motion of the ankle haptic platforms, the rotation angle is 0~25 degrees for dorsiflexion, 0~50 degrees for plantar flexion, as well as 0~32 degrees for inversion and eversion movements. Several small springs under each platform maintained the platform in a neutral position. Two highly adjustable lower-extremity supporters were aligned with each ankle haptic platform, which had an adjustable length and height of up to 60 cm and 70 cm, respectively. Each participant was asked to place their thighs on the lower extremity supporters and feet on the ankle haptic platforms which were fixed by Velcro to avoid potential abnormal compensatory movements in the coordination control tasks of the ankle joints. All movement data of the bilateral ankle joints were amplified, filtered using a low-level control box (custom made by Rmax Company, Taipei, Taiwan), and then collected and passed to a laptop through the NI DAQ system (USB-6003 Multifunction I/O and NI-DAQmx, National Instruments, Austin, TX, USA). Finally, the movement data of the bilateral ankles were calculated and displayed using LabVIEW (2015 edition, National Instruments, Austin, TX, USA). The self-developed measurement panel for measuring bilateral ankle coordination control tasks was designed using LabVIEW to acquire data from the rotary potentiometers in both ankles. The sampling rate was set at 1 kHz. The degree of ankle movements in the bilateral ankle coordination control tasks is shown on the control panel on a 32-inch LCD screen in LabVIEW.

### Bilateral ankle coordination control tasks

**Fig. 2:**
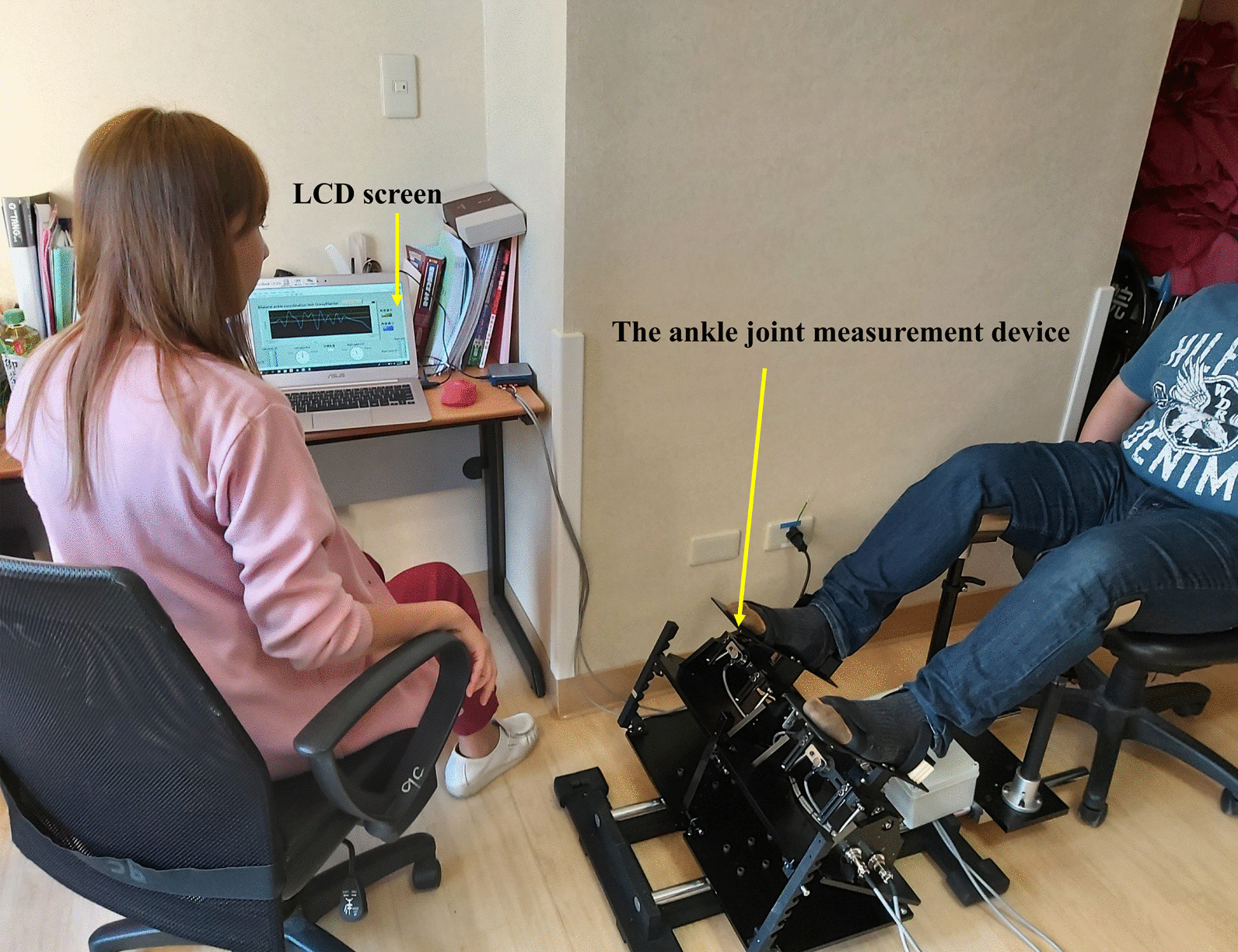
Experimental setting when performing bilateral ankle coordination control tasks

Based on the biomechanical analysis of gait performance, coordination control between bilateral ankles is an essential factor for reciprocal contact and lifting off the ground simultaneously, and causes body weight to smoothly shift from one leg to the other during walking [28, 29]. Therefore, the bilateral ankle coordination control tasks were mainly used to mimic gait performance requiring reciprocal coordination control of movement between two ankles. Before starting this task, participants were asked to place their dominant (non-paretic) and nondominant (paretic) foot on each platform fixed by Velcro and position their feet in a natural position. Then, one ankle was positioned at the dorsiflexion 10° position (positive number in degrees, + 10°), and the other ankle was positioned at the plantar flexion 10° position (negative number in degrees, − 10°) simultaneously. When the researcher gave the oral instruction “start,” the participants began to slowly move the ankle in dorsiflexion 10° to the plantarflexion 10° position, while the other ankle simultaneously moved from plantarflexion 10° to the dorsiflexion 10° position. Condition 1 was defined as reciprocal coordination control from 10° dorsiflexion to 10° plantarflexion in the dominant/non-paretic ankle and simultaneous reciprocal coordination control from 10° plantar flexion to 10° dorsiflexion in the non-dominant/paretic ankle in healthy subjects and patients with stroke. Condition 2 was the opposite for both healthy subjects and patients with stroke. The average degree of both ankles was displayed on the 32-in. LCD screen during each task; providing visual feedback to participants about whether both ankles were coordinated and close to the target degree (zero degree). The instructions given to each participant were: “The monitor provides the average degree of both ankles, please keep the average degree of both ankles close to the target degree when you switch the movements from one foot to the other smoothly during the task. Take your time; there is no time limit”. Each subject performed the task at their own speed. One practice round was performed to familiarize each participant with the coordination control task. The movement in degrees and alternation time between the two ankles were collected and evaluated (Fig. 2).

### Outcome measurements for bilateral coordination control of both ankles

The “alternating time” and “alternating angle” values for coordination control with the movements of the two ankles were calculated. Referencing previous studies to indicate coordination control between bilateral limbs [23, 24], the value of the alternating time for coordination with the movement of two ankles was calculated and defined as the operation time beginning from dorsiflexion/plantarflexion of the movement with one ankle to the cross point of bilateral movement of both ankles. A higher alternating time value indicated that the coordinated control of both ankles required a longer execution time. The value of the alternating angle for coordination with the movements of the two ankles was also calculated and changed into absolute values and defined as the coordination degree that was exerted by both ankles at the cross point when the ankle that was applying the dorsiflexion (plantarflexion) movement was changed to the plantarflexion (dorsiflexion) movement. If the value of the alternating angle is close to zero, it indicates better coordination control in bilateral ankles, which means that the participant could simultaneously and equally decrease or increase the dorsiflexion/plantarflexion movements between the bilateral ankles. To enable comparison of the alternating times for each subject, the data units were normalized as a percentage.

### Clinical motor and functional measurements

The lower extremity portion of the Fugl-Meyer assessment (FMA-LE) is the most commonly used clinical assessment tool for identifying motor recovery of the paretic lower limb in patients with stroke and was applied in this study [30–33]. Functional performance evaluations included the Berg Balance Test (BBS), Timed Up and Go Test (TUG), and Barthel Index (BI). The BBS is an excellent measurement tool with high reliability and can be used to measure static and dynamic balance in patients with stroke [34, 35]. The TUG test is also a reliable tool for assessing lower limb mobility in people after stroke [36, 37]. BI is one of the most common scales used in clinics to understand the independence of daily activities [38]. We also used a reliable and valid APDM inertial sensor system (APDM Inc., Portland, Oregon, United States) to quantitatively analyze the gait variables during the 10-m walking test [39], including walking speed, stride frequency, step length, and time in paretic and nonparetic legs in patients with stroke.

### Statistical analysis

Paired sample t-tests were used to analyze differences in the alternating time and angle for coordination with the movements of the two ankles between the two conditions for the healthy and stroke groups. The normality of these data was evaluated using the Shapiro–Wilk test, and the independent sample t-test was used to analyze the differences in the alternating time and alternating angle of both values between the groups. Relationships between the values of alternating time and alternating angle, the scores of the FMA-LE, BBS, TUG, and BI, and the gait variables of 10-m walking were analyzed using Spearman’s correlation coefficients. The alpha level of statistical significance was set at 0.05. The statistical software used was the SPSS ver. 19.0 (IBM Corporation, Armonk, NY, USA).

## Results

### Both ankles performance during the coordination control task

**Fig. 3:**
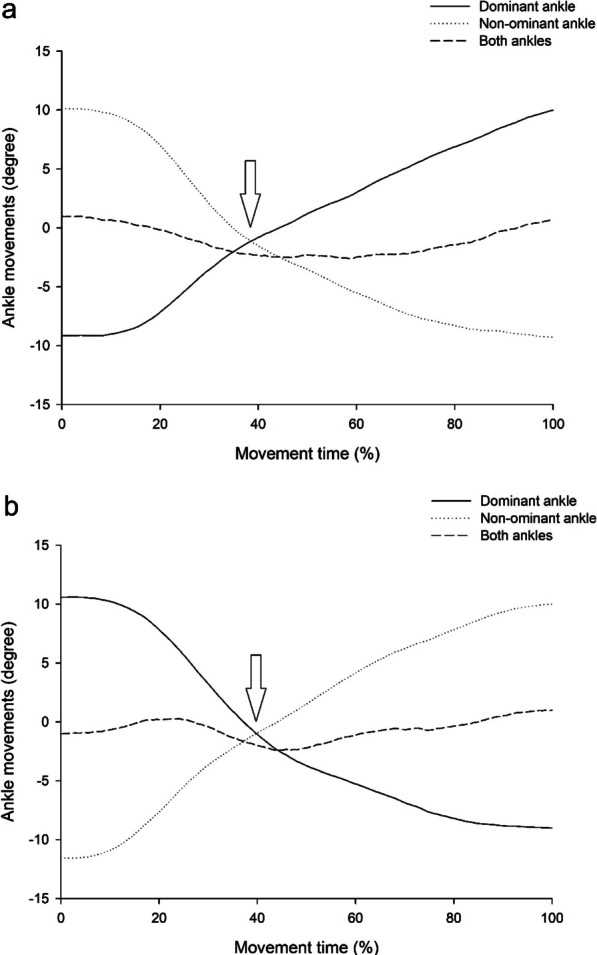
Representative plot of ankle movements in both ankles during coordination control tasks of the healthy group. The arrows indicate the cross point of the bilateral ankle movement coordination control tasks

**Fig. 4:**
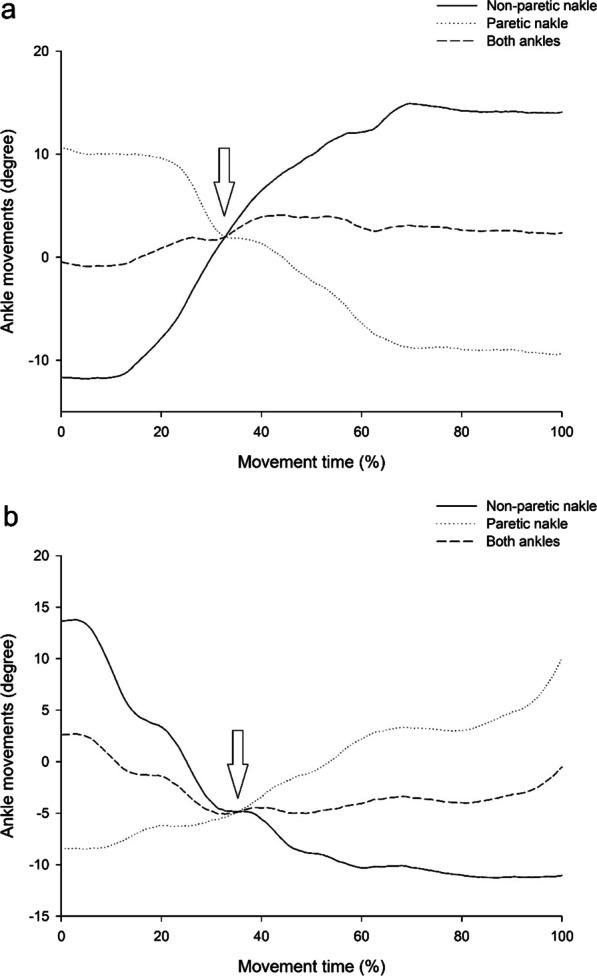
Representative plot of ankle movements in both ankles during coordination control tasks of the stroke group. The arrows indicate the cross point of the bilateral ankle movement coordination control tasks

Figures 3 and 4 provide representative plots of ankle movement performance during the coordination control task in the healthy and stroke groups, respectively. Arrows indicate the cross-point of the bilateral ankle movement coordination control task. The average degree of both ankles at the cross point was found to be close to zero (− 1° to − 1.1°) during the coordination control tasks of ankle movements from the dominant to the non-dominant ankle and vice versa in the healthy group (Fig. 3). Meanwhile, the SD values of the average degrees in both ankles during coordination control tasks were small (between 0.9° and 1.1°) during the coordination control tasks from one foot (dominant/non-dominant) to the other (non-dominant/ dominant)in healthy subjects (Fig. 3). These findings revealed that bilateral coordination control in the ankles is very stable and smooth. However, in the stroke group, the average degree of both ankles at the cross-point was found to deviate from zero (2.0° to − 4.8°) during the coordination control tasks of ankle movements from the paretic to the non-paretic ankle and vice versa (Fig. 4). Meanwhile, the SD values of the average degrees in both ankles during coordination control tasks were higher (between 1.5° and 2.1°) during the coordination control tasks from one foot (paretic/non- paretic) to the other (non- paretic/paretic) in stroke participants (Fig. 3). Additionally, compared with the non-paretic ankle, the movement of the paretic ankle was delayed and unable to quickly generate dorsiflexion movement of the coordination control of ankle movement from the non-paretic to the paretic ankle. Furthermore, a sudden rebound in ankle movement in the non-paretic limb was also found when moving the paretic ankle from a 10° plantarflexion position to 10° dorsiflexion during the coordination control task in stroke patients. These findings showed unstable bilateral coordination control in both the paretic and non-paretic ankles.

### Stroke-related changes in bilateral ankles coordination control performance

Compared with the healthy group, the alternating time was shorter from the non-paretic to the paretic ankle in the stroke group than from the dominant to the non-dominant ankle in the healthy group (p = 0.015) (Table 2). Additionally, the alternating angle by stroke subjects was also found to be significantly higher from the non-paretic to the paretic ankle (condition 1) and vice versa (condition 2) during the coordination control tasks than in the healthy group (p = 0.001 and p = 0.013, respectively) (Table 2). These findings indicate that patients with stroke have bilateral ankle coordination control deficits in spatial (degree) and temporal (time) coordination, and poorer coordination control in the paretic and non-paretic ankles in stroke patients.

**Table 2:** Comparison of bilateral ankles coordination control during non-synchronization tasks among the healthy and stroke groups

Coordination control variables	Conditions	Healthy group (*N* = 21)				Stroke group (*N* = 19)				Between groups	
		Mean ± SD	Difference	t	Sig (2-tailed)	Mean ± SD	Difference	t	Sig (2-tailed)	*F*	Sig (2-tailed)
Alternating time	Condition 1	43.7 ± 7.7	1.96 ± 10.47	.861	.399	35.4 ± 11.9	− 1.68 ± 15.87	− 0.464	.648	6.927	.012**
	Condition 2	41.7 ± 9.8				37.1 ± 13.5				1.544	.222
Alternating angle	Condition 1	1.0 ± 0.9	0.11 ± 0.66	.823	.420	2.5 ± 1.5	− 0.92 ± 2.61	− 1.537	.142	13.586	.001**
	Condition 2	0.9 ± 0.6				3.4 ± 2.4				21.394	.013**

Condition 1 was the coordination movement for the dominant (non-paretic) ankle from dorsiflexion 10° to plantarflexion 10° and simultaneously moving the non-dominant (paretic) ankle from plantar flexion 10° to dorsiflexion 10° in healthy and stroke subjects; Condition 2 was the coordination movement for the non-dominant (paretic) ankle from dorsiflexion 10° to plantarflexion 10° and simultaneously moving the dominant (non-paretic) ankle from plantar flexion 10° to dorsiflexion 10° in healthy and stroke subjects

*SD* standard deviation

^**^Significant difference p < 0.05

### Relationships between bilateral ankles coordination control and clinical motor and functional performances in the stroke group

The alternating angle from the paretic ankle to the non-paretic ankle was significantly correlated with BI scores (r =  − 0.475; p = 0.049), TUG (r = 0.512; p = 0.025), and 10-m walking (r = 0.747; p < 0.001) (Table 3). The alternating angle from the paretic ankle to the non-paretic ankle was moderately correlated with gait speed (r =  − 0.495, p = 0.031; r =  − 0.491, p = 0.033) and step length (r =  − 0.551, p = 0.015; r =  − 0.518, p = 0.023) in paretic and non-paretic ankles, respectively (Table 3).

**Table 3:** Relationships between bilateral ankles coordination control and clinical motor and functional performances in the stroke group (N = 19)

Coordination control variables	Conditions	Statistic	BI	FMA-LE	BBS	TUG (s)	10-m walk (s)	Cadence		Speed		Length		SI	
								P	NP	P	NP	P	NP	P	NP
Alternating time	Condition 1	Pearson correlation	− .017	.438	− .192	.059	− .062	.170	.169	.162	.184	.068	.127	.055	.070
		Sig. (2-tailed)	.945	.061	.432	.810	.802	.487	.489	.508	.451	.783	.604	.824	.776
	Condition 2	Pearson correlation	.169	.384	.143	− .390	− .220	.387	.387	.331	.346	.247	.291	.181	.422
		Sig. (2-tailed)	.489	.105	.560	.098	.365	.102	.101	.167	.147	.308	.226	.457	.072
Alternating angle	Condition 1	Pearson correlation	− .216	− .393	− .264	− .003	.106	− .276	− .280	− .251	− .235	− .145	− .114	.189	− .257
		Sig. (2-tailed)	.375	.096	.274	.990	.666	.252	.246	.300	.332	.554	.641	.437	.287
	Condition 2	Pearson correlation	− .457*	− .308	− .335	.512*	.747**	− .433	− .432	− .495*	− .491*	− .551*	− .518*	− .314	− .451
		Sig. (2-tailed)	.049	.199	.161	.025	.000	.064	.065	.031	.033	.015	.023	.191	.053

Condition 1 was transferred from the non-paretic to the paretic ankle in a stroke patient; condition 2 was transferred from the paretic to the non-paretic ankle in a stroke patient

*SD* standard deviation; *FMA-LE* lower limb Fugl-Meyer Assessment, *BI* Barth Index, *BBS* Berg Balance Test, *TUG* time up and go, SI symmetry index, *NP* Non- paretic, *P* paretic

^*^Significant difference p < 0.05, **p < 0.00

## Discussion

### Unstable bilateral ankle movements during bilateral coordination control tasks in stroke patients

Ankle movement is an important fundamental capability for performing and maintaining balance, posture, and walking in daily activities [40, 41], especially in ADLs requiring fast and precise reciprocal coordination control of bilateral ankle joint movements during walking and turning [42]. In this study, we used a novel bilateral ankle measurement system to collect movements in the degree of bilateral ankles and quantitatively measured coordination control of the bilateral ankles. The results showed that bilateral coordination control in healthy subjects was stable and they smoothly performed reciprocal ankle coordination movements with small deviations during the coordination control tasks (Fig. 3). In contrast, poorer bilateral coordination control with large deviations in both paretic and non-paretic ankles was found along with delayed and slow movement responses in the paretic ankle in patients with stroke (Fig. 4). Poor bilateral ankle coordination control could result from spasticity, weak muscle strength, joint stiffness, and proprioceptive deficits in the paretic ankle in people after stroke [43–48]. An earlier study also reported longer reaction times in muscle activation for the paretic limb than for the other non-paretic limb in patients with stroke [49]. In addition, a sudden rebound in ankle movement in the non-paretic limb was also observed when moving the paretic ankle from plantarflexion to dorsiflexion in patients with stroke. The potential neural mechanisms for the sudden rebound in ankle movement in the non-paretic limb could be that the ipsilateral corticospinal neurons were induced from the unaffected hemisphere, and then facilitated the activity in the non-paretic ankle in patients with stroke [50, 51]. In clinical observations, we also found that the gait pattern in the non-paretic limb was affected (sudden shaking of ankle movement) when patients started to move the paretic limb for several stroke patients during walking. This could result from the paretic limb starting to move the ankle to dorsiflexion position at the initial swing phase, and then causing a sudden rebound in ankle movement in the non-paretic limb in stroke patients. This phenomenon is also commonly observed in Brunnstrom stage 3 stroke patients and needs further investigation into the relationship between the rebound reaction in ankle movement in the non-paretic limb and gait performance in future studies. These bilateral ankle coordination control deficits were first revealed in this study and are rarely discussed in previous studies that used the bilateral limb coordination control tests of FMA and LEMOCOT in stroke patients [17, 18, 21, 22], which could lack appropriate measurement tools to quantitatively evaluate the coordination control of bilateral ankles in stroke patients.

### Differences in the bilateral ankle movement coordination control for the healthy and stroke groups

For excellent coordination control of the bilateral ankles, the alternating angle should be close to zero degree; this means that when one foot moves to dorsiflexion/plantarflexion to one degree, the other foot should follow the one degree movement in the opposite direction simultaneously during the coordination control task. For example, the healthy group exhibited alternating angles of 1.0 ± 0.9° and 0.9 ± 0.6° under conditions 1 and 2, respectively. However, the results indicated that the alternating angles were also significantly higher in the stroke than in the healthy group under conditions 1 and 2, by 1.5° and 2.5°, respectively (Table 2). Meanwhile, the alternating time was significantly shorter from the non-paretic to the paretic ankle in the stroke group than from the dominant to the non-dominant ankle in the healthy group by 8.3% (Table 2). The increasing alternating angles from the paretic to the non-paretic and non-paretic to the paretic ankles in the stroke group were not surprising because these values could be influenced by muscle weakness and poorer perception of the paretic ankle [9, 48]. However, compared with the dominant to non-dominant ankle (condition 1) of the healthy group, the alternating time of bilateral ankle movement coordination from the non-paretic to paretic ankle was shorter in patients with stroke. This finding was surprising because we expected that the alternating time from the non-paretic to the paretic ankle could be longer because the paretic ankle was delayed and unable to generate dorsiflexion movement quickly. This phenomenon could have resulted from a compensatory reaction in the paretic limb; a recent study reported that compensatory behavior in the paretic limb could be induced by poorer muscle strength generation and modulation of the paretic limb, leading to a decrease in alternating time during coordination control of both limbs [24]. In addition, the ipsilateral corticospinal pathways from the unaffected hemisphere could be facilitated by movement in the paretic limb [50, 51], and result in a shorter alternating time for the paretic ankle from plantar flexion to dorsiflexion in the stroke compared to the healthy group [50, 51]. Meanwhile, most patients with stroke had alternating angles in degrees at the cross point in dorsiflexion positions when performing the coordination control tasks in both conditions in the stroke group, which means the stroke patients focus on quickly increasing the dorsiflexion movement rather than the plantarflexion movement in both paretic and non-paretic ankles. This is a reasonable explanation for the alternating angles in degrees at the cross point in dorsiflexion positions because many studies focus on enhancing the movements of dorsiflexion in paretic ankles for the drop foot and prevent falling [52, 53] and the non-paretic ankle could be trained and affected as well. In contrast, clinical assessments (FMA-LE, BBS, and LEMOCOT) only provide scores to validate the coordination in the lower limbs by calculating the repetitions and times [21, 32, 54] rather than show the specific coordination disorder in movements. This study applied a novel bilateral ankle measurement device by combining the biosensors to calculate and provide alternating time and angle indices to calculate quantitative bilateral ankle coordination control deficits in spatial (degree) and temporal (time) domains, and revealed poorer coordination control in patients with stroke.

### The relationship between bilateral ankles coordination control and clinical motor and functional performances in the stroke group

We found a positive correlation between alternating angle for reciprocal coordination control under condition 2 with TUG and 10-m walk. This suggests that the alternating angles were increased when moving the paretic ankle from dorsiflexion to plantarflexion and moving the non-paretic ankle from plantar flexion to dorsiflexion simultaneously is associated with the TUG and 10-m walk tests in stroke patients. This indicates that imprecise coordination control can significantly increase the movement time of TUG and lead to poor walking performance in the lower limbs of patients with stroke. Furthermore, the results also showed a negative correlation of alternating angle under condition 2 with gait speed, length, and BI scores, which suggests that a higher alternating angle from dorsiflexion to plantarflexion in the paretic ankle and simultaneously moving the non-paretic ankle from plantar flexion to dorsiflexion is associated with slow gait speed, decreased step length, and quality of life in the BI score for patients with stroke. These findings were not surprising because a small alternating angle indicates better coordination control in bilateral ankles, and we expected that these poorer motor and functional performances would be associated with a larger alternating angle during the coordination control task. Meanwhile, a recent study also reported that coordination-related perception in paretic ankles is correlated with function in the lower limbs of stroke patients [48].

### Study limitations

This study had several limitations. Age and sex status were not matched between the stroke and healthy groups. The average age of the stroke group was 58.7 years, with four patients over 65 years old; therefore, the potential effect of aging on coordination control could have affected the results in this study [23]. Furthermore, differences in lesion sides of the brain related to coordination control could result in coordination deficit between both ankles of patients after stroke, which, however, we did not analyze and future study is suggested. Another limitation was that the number of participants recruited was affected by the COVID-19 pandemic, and larger samples may be required to draw firm conclusions in future research. Additionally, we indicated that the bilateral ankle coordination control deficits temporally and spatially by analyzing the alternating time and degree indexes and revealed poorer coordination control in stroke patients, which is a necessity for further studies to establish the psychometric properties (reliability, responsiveness) for these indexes of this method.

## Conclusions

Stroke results in unstable and poor bilateral ankle coordination control when starting movement of the paretic foot from plantar flexion to dorsiflexion, and vice versa. This study also demonstrated that a larger alternating angle between the non-paretic and paretic ankles was correlated with poor motor and functional performance in patients with stroke. We suggest that future rehabilitation programs should focus not only on motor and functional recovery for the paretic limb, but also on coordinative movement control training in both paretic and non-paretic limbs to improve functions in lower limbs of stroke patients.

## Data Availability

All data are available from the corresponding author upon reasonable request.

## Abbreviations

BI: Barthel Index
BBS: Berg Balance Test
FMA-LE: Fugl-Meyer assessment
LEMOCOT: Lower Extremity Motor Coordination Test
TUG: Timed Up and Go Test
